# Lycopene-Mediated Modulation of Apoptosis and Survival Signalling in Cancer: Emerging Nanodelivery Strategies and Translational Limitations

**DOI:** 10.3390/pharmaceutics18101214

**Published:** 2026-09-24

**Authors:** Ajay Kumar, Nidarshana Chaturvedi Parashar, Gaurav Parashar, Isha Rani, Damandeep Kaur, Sandeep Sharma, Hardeep Singh Tuli, Gurpreet Kaur Bhatia, Tarique Sarwar, Arshad Husain Rahmani

**Affiliations:** 1 Department of Environmental Science, Graphic Era (Deemed to be University), Dehradun 248002, Uttarakhand, India; kumar.ajay1250@gmail.com; 2 Department of Life Sciences, Parul Institute of Applied Sciences, Parul University, Vadodara 391760, Gujarat, India or nidarshana.parashar40880@paruluniversity.ac.in; 3 Division of Biomedical and Life Sciences, School of Science, Navrachana University, Vadodara 391410, Gujarat, India; 4 Department of Biochemistry, RIMT Medical College and Hospital, Mandi Gobindgarh 147301, Punjab, India; 5 University Center for Research & Development (UCRD), Chandigarh University, Mohali 140413, Punjab, India; 6 Centre for Research Impact and Outcome, Chitkara University, Rajpura 140417, Punjab, India; 7 Division of Research and Development, Lovely Professional University, Phagwara 144411, Punjab, India; 8 Centre of Excellence in Computational Research and Drug Discovery, Department of Bio-Sciences and Technology, Maharishi Markandeshwar Engineering College, Maharishi Markandeshwar (Deemed to Be University), Ambala 133207, Haryana, India; 9 Department of Physics, Maharishi Markandeshwar Engineering College, Maharishi Markandeshwar (Deemed to be University), Ambala 133207, Haryana, India; gurpreet1308@gmail.com; 10 Department of Medical Laboratories, College of Applied Medical Sciences, Qassim University, Buraydah 51452, Saudi Arabia

**Keywords:** lycopene, apoptosis, cell signalling, synergistic effects, apoptosis, autophagy, cancer, nanodelivery, pharmacokinetics

## Abstract

Lycopene is a dietary carotenoid with anti-inflammatory, proapoptotic, antiproliferative, and antioxidant properties in cancer models. Its application is limited by poor solubility, inconsistent bioavailability, formulation challenges, and unclear mechanisms of action. This review explores the mechanistic, preclinical, and clinical evidence of lycopene’s involvement in cell death and survival pathways, considering experimental models, mechanistic specificity, relevance, pharmacokinetics, and clinical validation. The most supported mechanism is mitochondrial apoptosis, indicated by a higher BAX/BCL-2 ratio, mitochondrial disruption, cytochrome c release, and caspase activation. Evidence on autophagy is mixed, with no proof that it causes cancer cell death. Human studies are limited and heterogeneous, primarily using tomato products or purified lycopene, and have demonstrated exposure and tolerability but not anticancer effects. One phase I trial found that synthetic lycopene was well tolerated, with some pharmacokinetic and biomarker changes but showed limited efficacy when combined with docetaxel. In vitro, nanocarriers enhance lycopene’s dispersion, stability, and tumour targeting, although data on pharmacokinetics, biodistribution, manufacturing, and safety remain limited. Currently, lycopene is an investigational agent for cancer prevention rather than an approved therapy; existing conclusions relate to other uses. Progressing towards clinical application requires developing standardised formulations, confirming exposure in models, and designing appropriate clinical trials.

## 1. Introduction

Cancer constitutes a critical public health problem and is defined by the deregulation of signalling networks and a failure to regulate cell proliferation, angiogenesis, metastatic progression, and programmed cell death [1]. Conventional therapies include chemotherapy and radiotherapy, but their effectiveness may be limited by off-target toxicity, the development of treatment resistance, and poor clinical outcomes in advanced disease [2,3]. There has also been growing interest in naturally derived bioactive compounds that may serve as chemopreventive agents or therapeutic adjuvants [4,5]. However, natural origin should not be interpreted as evidence of an absence of toxicity or established clinical efficacy. Lycopene is one such carotenoid. It is abundant in tomatoes and tomato products and is also present in several other red and pink fruits. Experimental studies suggest that lycopene may exert pleiotropic effects on cancer-cell proliferation and survival [6,7]. Lycopene is an acyclic hydrocarbon carotenoid with 11 conjugated and two non-conjugated double bonds [6,8]. Its extended polyene chain contributes to its reactivity toward reactive oxygen species and makes it an efficient singlet-oxygen quencher among biological carotenoids [6,9]. In addition to its antioxidant properties, lycopene has shown preclinical anticancer activity by modulating genes associated with cell-cycle regulation and inflammation, as well as key intracellular signalling cascades such as the PI3K/AKT/mTOR, Wnt/β-catenin, and MAPK/ERK pathways [4,7,10]. Several in vitro and animal studies have demonstrated that lycopene can influence apoptosis-related processes in cancer models. These effects include modulation of mitochondrial apoptosis, caspase activation, and expression of apoptosis-associated proteins; however, responses vary according to cancer type, experimental model, dose, and formulation [10,11,12]. Several studies reported apoptosis-associated responses following lycopene exposure, including increased pro-apoptotic BAX expression, reduced anti-apoptotic BCL-2 expression, mitochondrial membrane depolarisation, cytochrome c release, and caspase activation. However, these molecular events are not uniformly observed across all models and should be interpreted as context-dependent pathway responses rather than universal mechanisms [10,13,14]. Observational studies have examined associations of dietary lycopene intake, estimated from dietary assessments, and circulating lycopene concentrations measured in biological samples, with prostate cancer risk. However, these studies are observational in nature and cannot establish causal relationships or demonstrate therapeutic efficacy. Some studies have reported an inverse association between higher lycopene exposure and prostate cancer incidence or progression-related outcomes; however, these findings are observational and cannot establish causality or therapeutic efficacy [15,16]. The clinical translation of lycopene is hindered by its hydrophobicity, which limits aqueous solubility, as well as its chemical instability and variable absorption influenced by food matrix composition, dietary fat content, isomeric distribution, formulation, and host-related factors [17]. To overcome these physicochemical limitations, researchers have explored nanoscale delivery systems such as cubosomes, nanomicelles, liposomes, niosomes, and lipid-based carriers to improve lycopene dispersion, stability, uptake, and in vivo site-specific delivery [4,5,18]. However, these changes in formulation properties and/or experimental tumour targeting should be interpreted with caution as evidence of clinical efficacy. Lycopene has also been reported to make cells more sensitive to chemotherapeutic drugs such as 5-fluorouracil and cisplatin in cell culture and selected animal models by regulating apoptosis and the drug-resistance pathway. However, no clear evidence has shown that lycopene enhances chemotherapy effectiveness and/or reduces chemotherapy-related systemic toxicity in patients [3,5]. The biological effects of lycopene depend on multiple context-dependent factors, including cellular identity, tissue origin, species background, molecular alterations within disease models, metabolic capacity, concentration, formulation, and exposure duration. Therefore, findings from one experimental model should not be directly extrapolated to other biological systems without accounting for these differences. This review interprets evidence in the context of each experimental design. This review assesses the mechanisms of lycopene-induced regulated cell death, distinguishes findings from in vitro (cell culture) and in vivo (animal models and human biomarkers) studies, and identifies the pharmacokinetic and methodological requirements that must be met before justifying lycopene as a nutrition-based therapeutic strategy. The definition of “anticancer activity” throughout this review indicates any activity that relates to an experimental molecular, cellular or tumour-related endpoint unless human clinical activity is specifically cited.

## 2. Methodology

The present narrative review was developed using a pre-defined literature search and evidence synthesis process to review pertinent studies on the published evidence of lycopene’s anticancer activity, with particular attention drawn to the mechanisms of regulated cell death, modulation of cancer survival signalling pathways, nanodelivery systems, chemosensitisation, pharmacokinetics and pharmacogenetics, safety considerations, and clinical translation. We searched PubMed/MEDLINE, Scopus and Web of Science Core Collection from database inception, using Boolean operators to “OR” terms, and modified terms to match database indexing. The main search strategy was “lycopene” AND “cancer” AND (combinations of: “apoptosis”, “programmed cell death” OR “Bcl-2/Bax”, “Fas-FasL”, “caspase activation”, “PI3K/Akt/mTOR”, “JAK/STAT3”, “autophagy”, “PANoptosis”, “nanodelivery”, “bioavailability”, or “chemosensitization”). Eligible publications consisted of original in vitro, in vivo animal, pharmacokinetic, human observational, controlled and uncontrolled clinical intervention, formulation, and toxicological reports that provided insight into mechanisms, pharmacologic evaluations, safety studies, or translation of lycopene against cancer prevention or cancer therapeutics. We also identified secondary studies, including review articles for background concepts and other primary studies for therapeutic efficacy but did not use them as primary evidence of efficacy. We prioritised studies from the last five years and included seminal studies that provided key mechanistic, pharmacokinetic, or clinical data whose significance had not been undermined by newer studies. Studies that did not have relevance to cancer biology, did not measure a lycopene intervention or a well-described lycopene-containing intervention, did not include adequate methodological information to interpret the results, reported computational predictions but not experimental measures, were duplicate publications, or did not include outcome measures relevant to regulated cell death, cancer-survival signalling, pharmacokinetics, delivery, safety, or human translation, were excluded. Only conference abstracts, editorials, letters (no data) and non-peer-reviewed items were excluded. The selected literature was critically and thematically reviewed to synthesise current knowledge on lycopene’s role in regulating cell death, modulating survival-signalling pathways, pharmacokinetic limitations, lycopene formulation development, combination therapies, safety, and translatability.

## 3. Chemistry, Structure–Activity Relationship, and Pharmacokinetics

Lycopene (C_40_H_56_) is a linear, acyclic hydrocarbon carotenoid, which is symmetrical as shown in Figure 1 [1,2]. It lacks terminal ionone rings that human systems can convert to vitamin A, so it is not considered a carotene [3,4]. It has 11 conjugated and two non-conjugated double bonds. The ability of the extended conjugated system to provide efficient singlet oxygen quenching has been reported to be around two times higher than the singlet oxygen quenching ability of β-carotene [3,4,5]. The geometrical configuration of lycopene plays a crucial role in relation to its structure–activity relationship. In nature, lycopene is found mainly in the all-trans configuration; however, cis-isomers (e.g., 5-Z, 9-Z, 13-Z) show substantially greater aqueous solubility and incorporation into mixtures of micelles and have higher tissue accumulation [6,7]. Oxidised lycopene derivatives, such as apolycopenals and lycopenoic acids, have electrophilic properties similar to those of lycopene and thus may contribute to biological activities, including activation of antioxidant-response signalling and modulation of NF-κB [8,9,10]. Lycopene is highly hydrophobic and has been reported to be poorly absorbed after oral administration, with bioavailabilities ranging from 10 to 30% depending on the food matrix, formulation, the isomeric composition of the lycopene consumed, and host-related factors (Figure 1) [8,11,12]. Food presence affects its absorption. Mechanical processing (e.g., homogenization) and/or thermal treatment (e.g., cooking in oil) improves bioavailability by breaking down cell walls and producing bioavailable cis-isomers [13,14,15]. Absorption occurs in the small intestine via passive diffusion and protein-mediated transport, specifically involving scavenger receptor class B type I (SR-BI) [16,17]. Post-absorption, lycopene is packaged into chylomicrons and transported through the lymphatic system into the bloodstream, where it is primarily associated with low-density lipoproteins (LDL) [17,18]. Although all-trans lycopene predominates in many dietary sources, cis-isomers are relatively enriched in plasma and tissues, potentially because of differences in absorption, postabsorptive isomerisation, transport, and metabolic stability (Figure 1) [19,20]. Its reported apparent plasma half-life is approximately 2–3 days, whereas retention may be longer in selected tissues [4,21,22]. Preferential tissue distribution may influence organ-specific exposure; however, it does not, by itself, establish site-specific anticancer efficacy [4,23].

### 3.1. Apoptotic and Survival Signalling Modulation in Cancer

Apoptosis is a highly regulated form of programmed cell death that occurs in response to cellular stresses, including DNA damage, oxidative imbalance, oncogenic stress, and metabolic disturbances. It proceeds primarily through the extrinsic death receptor pathway and the intrinsic mitochondrial pathway. Experimental studies suggest that lycopene can modulate components of both apoptotic routes while also affecting survival-signalling networks involved in proliferation, treatment resistance, and evasion of cell death. However, the strength of evidence varies substantially by experimental model, lycopene concentration, formulation, exposure duration, and endpoint examined (Table 1a–c).

### 3.2. Fas–FasL Pathway Activation

The extrinsic apoptotic pathway is initiated by ligand interaction with death receptors such as tumour necrosis factor (TNF)-related apoptosis-inducing ligand (TRAIL) receptor (TRAILR) and FAS/CD95/APO-1 [24]. The binding of Fas ligand to the Fas receptor promotes receptor trimerization, recruitment of the adaptor protein FADD, and formation of the death-inducing signalling complex. This complex activates initiator caspases-8 and -10, which subsequently activate effector caspases-3 and -7, culminating in apoptotic cell death [25]. In cancer models, lycopene has been shown to enhance FasL-mediated apoptotic signalling. Lycopene combined with eicosapentaenoic acid inhibited Akt activation and mTOR signalling, increased Fas ligand accumulation, and reduced the survival of HT-29 human colon cancer cells [26]. In addition, the lycopene auto-oxidation metabolite (E,E,E)-4-methyl-8-oxo-2,4,6-nonatrienal has been reported to induce Fas ligand expression and initiate apoptosis in HL-60 human promyelocytic leukaemia cells [27]. Caspase-8 activation may also cleave Bid to truncated Bid, thereby linking Fas–FasL-mediated extrinsic apoptosis with mitochondrial apoptotic signalling. Overall, lycopene seems to have a context-dependent effect on Fas–FasL signalling, though membrane-bound FasL is generally more effective in inducing apoptosis than soluble FasL. It may vary by cancer type, dose, formulation, and cellular redox status. Therefore, Fas–FasL activation should be considered an important component of lycopene-induced apoptosis rather than a universal mechanism across all malignancies.

### 3.3. Modulation of the Bcl-2/Bax Pathway

#### 3.3.1. Lycopene-Alone Effects on Mitochondrial Apoptosis

Numerous studies have shown that lycopene induces apoptosis via the intrinsic pathway. The intrinsic pathway involves permeabilisation of the mitochondrial outer membrane, leading to the cytosolic release of pro-apoptotic proteins, such as SMAC/Diablo and cytochrome c (Cyt c), from the intermembrane space. Cyt c binds to Apaf-1 protein and thereby activates caspase-9, which finally activates executioner caspases (caspases-3, -6, and -7) of apoptosis [28]. The intrinsic apoptotic pathway is regulated by pro-apoptotic (Bax, Bim, and Bid) and anti-apoptotic (Bcl-XL and Bcl-2) members of the Bcl-2 family, which regulate mitochondrial cytochrome c release. Synergism between lycopene and eicosapentaenoic acid (EPA) has been reported to inhibit mammalian target of rapamycin (mTOR), which, in turn, triggers Bax accumulation and hinders the survival of HT-29 human colon cancer and oral cancer cells [26,29]. In HeLa cervical cancer cells, treatment with the study-defined IC_50_ concentration of lycopene increased the expression of cleaved caspase-3, cleaved PARP, and BAX [30]. Lycopene has been reported to suppress proliferation and alter cell-cycle progression in human prostate cancer cells, suggesting effects on growth-regulatory mechanisms in prostate cancer models [31]. Furthermore, lycopene has been reported to increase BAX and decrease BCL-2 expression in HeLa cervical cancer cells and SKOV3 ovarian cancer cells [32,33]. In AGS gastric cancer cells, lycopene reduced cellular viability, induced DNA fragmentation, and increased the BAX/BCL-2 ratio [34]. In contrast, the synergistic effect of S-allylcysteine C and lycopene also decreased the Bcl-2/Bax ratio and upregulated Bim and caspases-8 and -3 [35]. Both low- and high-dose lycopene supplementation can substantially reverse the phosphorylation of BAD (Ser136 and Ser112) and reduce apoptosis by deactivating caspase-3 in squamous metaplasia induced by cigarette smoke-exposed ferrets [36]. Lycopene was observed to chemosensitize human colon cancer Caco-2 cells to 5-FU treatment by upregulating the Bax/Bcl-2 ratio and activating caspase-3 and caspase-9 gene expression [37]. Moreover, lycopene promotes apoptosis in a dose-dependent manner, increases Bax levels, and decreases Bcl-2 levels in pancreatic cancer PANC-1 cells [38]. A recent study has also shown that lycopene (7.89 μM) exhibits a pro-apoptotic effect by significantly elevating cleaved caspase-3 (*p* < 0.01) and Bax (*p* < 0.05) in the HT-29 colon cancer cell line, thereby exhibiting its potential as a useful compound in reducing the incidence of colon cancer [39]. Moreover, lycopene has been shown to induce mitochondrial apoptosis by increasing tBid expression and decreasing Akt phosphorylation in canine osteosarcoma cells [40].

#### 3.3.2. Combination Strategies Involving Lycopene

A recent in vivo study has also revealed that combined supplementation with lycopene and genistein significantly reduced breast cancer growth and development, with decreased Bcl-2 expression, increased Bax expression, and activation of caspases-3 and -9 [41]. Collectively, lycopene-mediated regulation of the Bcl-2/Bax axis is among the most consistent mechanisms underlying its pro-apoptotic activity in cancer models.

### 3.4. Targeting the PI3K/Akt/mTOR Pathway

The PI3K/AKT/mTOR pathway is a conserved signalling cascade in eukaryotic cells that regulates processes such as cell survival, growth, progression and angiogenesis. Alterations in this pathway, including PI3K overactivation, PTEN inactivation, or AKT gain-of-function mutations, as well as activation of anti-apoptotic factors, are well-established contributors to cancer development, disease progression, and resistance to therapy [41]. In in vitro and in vivo oral cancer models, lycopene treatment was reported to inhibit epithelial–mesenchymal transition and promote apoptosis by reducing PI3K/AKT/mTOR signalling [29]. In a combination-treatment study, lycopene together with eicosapentaenoic acid reduced Akt/mTOR activation and inhibited proliferation in HT-29 colon cancer cells [26]; because both agents were administered together, these molecular effects cannot be attributed exclusively to lycopene. In HT-29 human colon cancer cells, lycopene treatment suppressed Akt activation and reduced non-phosphorylated β-catenin levels, while increasing β-catenin phosphorylation and nuclear p27 abundance, changes associated with reduced cell proliferation [42]. Furthermore, lycopene triggered robust and sustained Erk1/2 activation, accompanied by cyclin D1 suppression and p21 upregulation in triple-negative breast cancer cells. In breast cancer cell models, lycopene inhibited AKT phosphorylation and downstream mTOR signalling and increased BAX expression [43]. These findings suggest that inhibition of PI3K/Akt/mTOR is a major survival-signalling mechanism through which lycopene promotes apoptosis and suppresses tumour progression.

### 3.5. JAK/STAT3 Signalling as a Context-Specific Target

Janus kinase (JAK)/signal transducers and activators of transcription (STAT) signalling integrates cytokine- and growth-factor-dependent signals with transcriptional programmes governing inflammation, proliferation, survival, and resistance to apoptosis [44]. However, direct evidence that lycopene supports JAK/STAT3 as a primary anticancer target remains limited. Natural compounds are considered potential JAK inhibitors because they can modulate cytokine-driven signalling. Few studies examine the effect of lycopene treatment on the JAK/STAT3 signalling pathway in cancer. The most directly relevant study used *Helicobacter pylori*-exposed human gastric epithelial cells and demonstrated that lycopene pretreatment attenuated reactive oxygen species-dependent JAK1/STAT3 and Wnt/β-catenin activation, together with reduced epithelial hyperproliferation [45]. Although this model is relevant to inflammation-associated gastric carcinogenesis, it represents a hyperproliferative gastric epithelial system rather than an established malignant-cell model or an in vivo therapeutic tumour model. Therefore, the study demonstrates pathway modulation in response to an inflammatory stimulus but does not establish JAK/STAT3 inhibition as a primary mechanism of lycopene-induced cancer cell death. Numerous studies are needed to validate the promising anticancer effects of lycopene by targeting the JAK/STAT3 signalling pathway during carcinogenesis. According to Yu et al. (2014) [46] and Hu et al. (2021) [47], lycopene also induces apoptosis in cancer cells by regulating the JAK-STAT3 signalling pathway. The JAK signalling pathway involves a cascade of protein interactions that transmit chemical signals from the cell interior to the nucleus, activating genes involved in cell proliferation, differentiation, survival, and apoptosis. JAK (Janus Kinase) is an intracellular protein kinase that becomes phosphorylated and forms dimers upon binding cytokines, followed by interaction with STAT (Signal Transducer and Activator of Transcription) proteins, which are translocated to the cell nucleus and ultimately regulate gene expression. Disruption of the JAK-STAT3 signalling pathway results in apoptosis resistance and cancer [46,47]. Phesse et al. demonstrated functional cross-talk between gp130–JAK–STAT3 and Wnt/β-catenin signalling during intestinal tumorigenesis [48]. However, that investigation did not evaluate lycopene. Thus, this study does not provide sufficient evidence for the biological relevance of the pathway interactions and should not be used to support the use of lycopene itself to inhibit JAK/STAT3. In a 2018 study, Park et al. examined gastric epithelial cells from humans infected with Helicobacter. ROS assay, Western blot, and proliferation assay were used to assess the effects of lycopene on ROS levels, activation, and cell proliferation, respectively, following cell pre-treatment. *H. pylori* increased ROS production, which activated the Jak1/Stat3 and Wnt/β-catenin signalling pathways, leading to excessive cell proliferation and cancer development [45]. Lycopene, by inhibiting Wnt/β-catenin signalling, may also explain reduced proliferation and increased susceptibility to cell death by interacting with other oncogenic pathways. However, additional in vivo and mechanistic investigations are needed to establish whether JAK/STAT3 is a primary or secondary target of lycopene across various cancer types. Evidence remains limited on its causal impact. So far, no consistent data show that JAK/STAT3 is a prerequisite for apoptosis, a common trait in multiple cancer types, or that genetic or pharmacological rescue or pathway-specific inhibition can counter it. In addition, the link between experimental dose and plasma or tumour exposure remains unclear. Based on the current evidence, JAK/STAT3 should probably be regarded as a potential, context-specific target rather than a universal target for lycopene’s anticancer activity. Future studies should evaluate direct target engagement, time dependence (STAT3 inhibition vs. cell death), genetic and/or pharmacological rescue of STAT3 inhibition, exposure–response relationships, and reproducibility in immune-competent animal tumour models.

### 3.6. Autophagy as a Context-Dependent Response

Autophagy, a dynamic degradation process in lysosomes, can act as either a double- or triple-agent in cancer. Under stress conditions and/or at basal levels, autophagy may remove damaged proteins and organelles to promote cell survival. In contrast, unregulated, incomplete, or impaired autophagic flux may contribute to cell dysfunction and cell death [49]. Thus, alterations in autophagy-associated proteins following lycopene treatment cannot be considered markers of lycopene-induced cancer-cell death via the autophagic pathway. Lycopene has been shown to regulate autophagy in a type-specific fashion, playing a role in tumour cell suppression across various cancer types through several identified mechanisms. Lycopene triggers apoptosis in HeLa cells by increasing levels of cleaved caspase-3, PARP, and Bax, and enhances autophagy by elevating LC3β expression, while also increasing oxidative DNA damage. In oral cancer cell lines (CAL-27, SCC-9), lycopene inhibits proliferation and epithelial–mesenchymal transition by blocking the PI3K/AKT/mTOR pathway at suitable concentrations. Additionally, it induces both autophagy and apoptosis in these cells [29]. Another study showed that lycopene blocked autophagic flux in COLO-16 cells via the mTORC1 pathway, thereby increasing activation of the ERK and JNK pathways. This modulation is linked to changes in tight junction protein expression, with ZO-1 upregulated and claudin-1 downregulated, thereby decreasing cell migration [50]. Additionally, lycopene could play a cytoprotective role in normal or precancerous cells by inducing autophagy (as evidenced by increased LC3-II expression and decreased p62 expression). This mechanism removes damaged cellular components and ROS, helping maintain genome integrity [51]. These seemingly contradictory results could reflect differences in cell origin, malignancy, lycopene content, treatment duration, formulation, oxidative environment, or the specific point of autophagic flux investigated. Therefore, avoid statements linking their beneficial effects on autophagy to anti-ageing, since they do not fit that characteristic. Static analysis of LC3-I/LC3-II, BECN1 or p62 is not enough to show autophagic flux or autophagy-related cell death. To provide more convincing evidence, include control experiments, including lysosomal inhibition; tandem fluorescent LC3 reporters; time-resolved flux measurements; and genetic or pharmacological modulation of key autophagy regulators, such as ATG5, ATG7, and BECN1.

### 3.7. PANoptosis and Other Regulated Cell-Death Pathways

PANoptosis refers to the coordinated engagement of apoptotic, pyroptotic, and necroptotic machinery through a multiprotein PANoptosome complex [52]. Demonstrating PANoptosis requires more than observing apoptosis alongside oxidative stress or changes in inflammatory signalling [53]. Convincing evidence should include activation of pathway-specific effectors, identification or assembly of PANoptosome components, and functional demonstration that perturbation of apoptotic, pyroptotic, and necroptotic machinery alters the cell-death phenotype [54]. The available lycopene literature does not currently provide direct evidence of PANoptosome formation or coordinated dependence on the three principal cell-death programmes [55]. Lycopene-induced caspase activation supports apoptosis in selected models, but apoptosis alone does not establish PANoptosis [56]. Similarly, changes in reactive oxygen species, inflammatory mediators, or autophagy-related markers cannot be used as substitutes for evidence of pyroptotic or necroptotic execution [57]. Direct evidence would require evaluation of pyroptosis-associated markers such as inflammasome activation and gasdermin cleavage, necroptosis-associated signalling such as RIPK3 and MLKL activation, apoptosis-associated caspase activity, and physical or functional interaction among PANoptosome components [58]. Genetic knockdown, pharmacological inhibition, and rescue experiments would also be necessary to establish coordinated pathway dependency. Evidence that lycopene protects normal tissues from ferroptosis, inflammatory injury, or oxidative damage should not be extrapolated to suggest that lycopene induces ferroptotic or PANoptotic death in cancer cells [59]. The biological effects of lycopene may differ substantially between malignant and non-malignant tissues [60,61]. PANoptosis, pyroptosis, necroptosis, ferroptosis, and related regulated cell death pathways should therefore be regarded as research questions in lycopene oncology rather than as established anticancer mechanisms (Figure 2). Currently, lycopene-specific evidence for PANoptosis is indirect and hypothesis-generating.

**Table 1:** (**a**) Apoptotic and survival-signalling modulation by lycopene and lycopene-derived interventions according to experimental context. (**b**) Lycopene-alone interventions and apoptosis/survival-signalling responses in experimental cancer models. (**c**) Combination interventions involving lycopene in experimental cancer models.

(**a**)						
**S. No.**	**Cancer Type/Model**	**Lycopene Intervention/Context**	**Intervention Category/Model**	**Reported Pathway/Protein Response**	**Key Apoptotic/Survival Effect**	**Reference**
1	HL-60 human promyelocytic leukaemia cells	Lycopene auto-oxidation metabolite MON ((E,E,E)-4-methyl-8-oxo-2,4,6-nonatrienal)	Lycopene-derived metabolite; in vitro mechanistic model	FasL, Bcl-2, Bcl-xL, caspase cascade	MON induced apoptosis through caspase activation, FasL induction, and downregulation of Bcl-2/Bcl-xL	[27]
2	HT-29 human colon cancer cells	Lycopene combined with eicosapentaenoic acid	Combination intervention; in vitro mechanistic treatment model	Akt/mTOR, FasL, Bax	Combined treatment reduced Akt/mTOR signalling, increased FasL and Bax, and suppressed cell survival; effects cannot be attributed exclusively to lycopene	[26]
3	LNCaP human prostate cancer cells	Physiologically attainable lycopene concentrations	Lycopene alone; in vitro mechanistic treatment model	Mitochondrial membrane potential, cytochrome c, Annexin V	Lycopene induced mitochondrial apoptosis and reduced prostate cancer cell proliferation	[62]
4	LNCaP and PC-3 prostate cancer cells	Lycopene-based products	Lycopene intervention; in vitro mechanistic treatment model	Apoptosis-associated and androgen-responsive signalling	LNCaP cells showed greater apoptosis-associated responses, whereas PC-3 cells were comparatively resistant	[63]
5	Human prostate cancer tissue; pre-prostatectomy study	Lycopene supplementation	Preoperative clinical biomarker-modulation model; not therapeutic efficacy	Bax, Bcl-2, connexin 43, IGF-related markers	Lycopene supplementation altered selected prostate cancer-related biomarkers; Bax and Bcl-2 changes were not definitive	[64]
6	Patients with prostate cancer consuming tomato sauce before prostatectomy	Dietary tomato-sauce lycopene exposure	Preoperative dietary biomarker/exposure model; not therapeutic efficacy	Apoptosis-associated markers, oxidative DNA damage	Tomato-sauce intake increased prostate tissue lycopene exposure and altered selected apoptosis- and oxidative-stress-related biomarkers	[65]
7	Multiple cancer cell lines	Lycopene treatment	Lycopene alone; in vitro mechanistic treatment model	Mevalonate/Ras signalling, NF-κB, cell-cycle proteins	Lycopene inhibited cancer-cell growth and altered proliferation-, cell-cycle-, and apoptosis-associated signalling	[66]
(**b**)						
**S. No.**	**Cancer Type/Model**	**Lycopene Intervention/Context**	**Intervention Category/Model**	**Reported Pathway/Protein Response**	**Key Apoptotic/Survival Effect**	**Reference**
1	Prostate and breast cancer cells	Physiologically relevant lycopene concentrations	Lycopene alone; in vitro mechanistic treatment model	IκB kinase, NF-κB signalling	Lycopene suppressed cancer-cell growth in association with inhibition of IκB kinase and NF-κB activation	[67]
2	SKOV3 ovarian cancer cells	Lycopene treatment	Lycopene alone; in vitro mechanistic treatment model	Bax, Bcl-2	Lycopene increased Bax expression, decreased Bcl-2 expression, and inhibited ovarian cancer-cell proliferation	[32]
3	AGS gastric cancer cells	Lycopene treatment	Lycopene alone; in vitro mechanistic treatment model	β-catenin, Bax/Bcl-2 ratio, ROS	Lycopene reduced cell viability, induced DNA fragmentation, increased the Bax/Bcl-2 ratio, and inhibited β-catenin signalling	[34]
4	AGS gastric cancer cells	Lycopene treatment	Lycopene alone; in vitro mechanistic treatment model	EGFR/Ras/MAPK, NF-κB/COX-2	Lycopene inhibited proliferation and promoted apoptosis-associated responses together with suppression of EGFR/Ras/MAPK and NF-κB/COX-2 signalling	[68]
5	PANC-1 pancreatic cancer cells	Lycopene treatment	Lycopene alone; in vitro mechanistic treatment model	ROS, NF-κB, Bax/Bcl-2, active caspase-3	Lycopene reduced ROS and NF-κB signalling while increasing the Bax/Bcl-2 ratio and active caspase-3	[38]
6	HT-29 colon cancer cells	Lycopene treatment	Lycopene alone; in vitro mechanistic treatment model	Bax, Bcl-2, cleaved caspase-3, DNA damage	Lycopene produced antiproliferative and apoptosis-associated effects in HT-29 cells	[39]
7	Oral cancer cells and experimental oral cancer model	Lycopene treatment	Lycopene alone; preclinical mechanistic/treatment model (in vitro and in vivo)	PI3K/Akt/mTOR, EMT markers, apoptosis	Lycopene inhibited EMT and promoted apoptosis in association with reduced PI3K/Akt/mTOR signalling	[29]
8	Human breast cancer cell lines	Lycopene treatment	Lycopene alone; in vitro mechanistic treatment model	Akt, mTOR, Bax, ERK1/2, cyclin D1, p21	Lycopene modulated proliferation- and apoptosis-associated Akt/mTOR and cell-cycle signalling	[43]
9	Canine osteosarcoma cells	Lycopene treatment	Lycopene alone; in vitro mechanistic treatment model	tBid, Akt phosphorylation, mitochondrial apoptosis	Lycopene induced apoptosis-associated mitochondrial signalling and reduced Akt phosphorylation	[40]
10	HeLa cervical cancer cells	Lycopene treatment	Lycopene alone; in vitro mechanistic treatment model	Cleaved caspase-3, cleaved PARP, Bax, LC3β	Lycopene induced apoptosis-associated changes together with modulation of autophagy-related markers	[30]
(**c**)						
**S. No.**	**Cancer Type/Model**	**Lycopene Intervention/Context**	**Intervention Category/Model**	**Reported Pathway/Protein Response**	**Key Apoptotic/Survival Effect**	**Reference**
1	HT-29 human colon cancer cells	Lycopene combined with eicosapentaenoic acid	Combination intervention; in vitro mechanistic treatment model	Akt/mTOR, FasL, Bax	Combined treatment reduced Akt/mTOR signalling, increased FasL and Bax, and suppressed cell survival; the effects cannot be attributed exclusively to lycopene	[26]
2	DMBA-induced breast carcinogenesis in rats	Lycopene plus genistein supplementation	Combination intervention; carcinogen-induced chemopreventive model	Bax, Bcl-2, caspase-3, caspase-9, oxidative stress	Combined supplementation reduced breast cancer development and altered apoptosis-associated proteins	[41]
3	Caco-2 colon cancer cells	Lycopene combined with 5-fluorouracil	Combination/chemosensitization intervention; in vitro treatment model	Bax/Bcl-2, caspase-3, caspase-9	Combined treatment enhanced apoptosis-associated responses and sensitised Caco-2 cells to 5-FU	[37]

**Figure 2:**
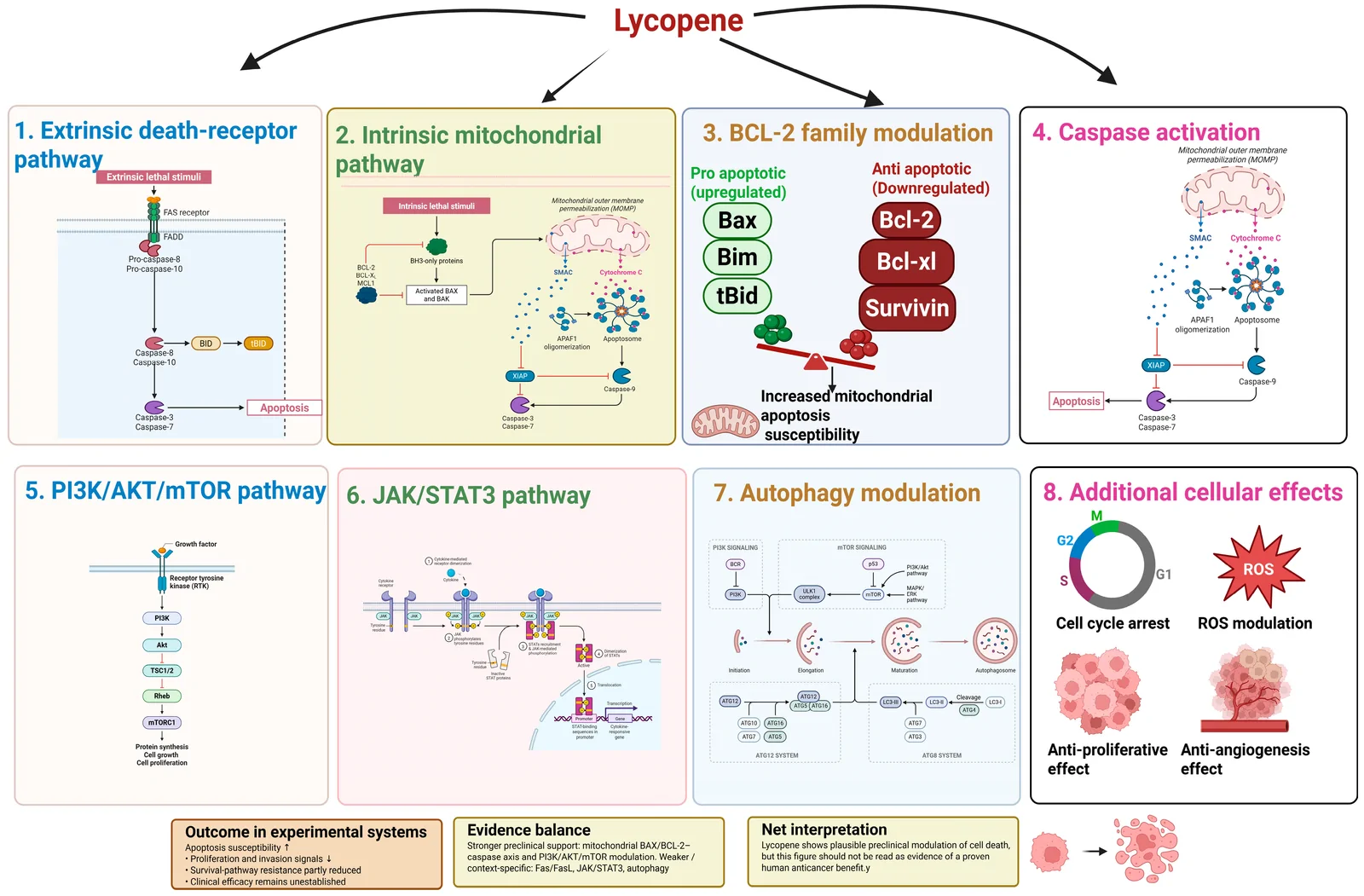
Lycopene: modulation of apoptotic and survival signalling pathways in cancer cells. Created in BioRender. RAHMANI, A. H. (2026) https://BioRender.com/8rpm5ub.

## 4. Human Evidence and the Translational Gap

Human evidence was evaluated separately because diet-related claims fall into several categories, including diet associations, biological exposure, tissue accumulation, tolerability, and clinical efficacy [69,70]. Most human studies conducted are small, short-term, prostate cancer or prostate cancer-related biomarker studies [71]. In contrast, short preoperative studies explore exposure markers or the feasibility of prevention hypotheses, while observational studies test prevention hypotheses without proving causality. Plasma and tissue levels do differ according to pharmacokinetic studies. In one preprostatectomy study, no more than 25–35 mg of lycopene raised plasma concentrations by 59–71% and led to a 2.2–3.6-fold increase in lycopene levels in prostate tissues after consumption with tomato soup and tomato sauce or juice, respectively, as compared to a control diet. There is also only a moderate correlation between plasma and tissue concentrations, so they do not necessarily match. Small trials with tomato oleoresin or foods have demonstrated improvements in tissue lycopene concentration and some biomarker changes, although not always statistically significant, with short exposure durations (typically 3–4 weeks) and no survival or recurrence data [64,72]. Controlled studies have shown mixed results. One trial did not find any changes in PSA in 79 men with prostate cancer given 30 mg lycopene daily for three weeks. Some effects observed in specific groups were exploratory and need to be confirmed [73]. Data for treatments involving chemotherapy is still preliminary. Another phase II trial of docetaxel in combination with 30 mg/day of lycopene in 13 men with metastatic prostate cancer reported survival and PSA improvement but failed to include a control group; therefore, it is not able to establish cause and effect. In a second phase I trial using synthetic lycopene, tolerable doses were determined, pharmacokinetic changes were observed, and there was no clear evidence of tumour response [74]. Future trials should carefully evaluate drug interactions that affect docetaxel pharmacokinetics and anticancer activity, as lycopene can alter docetaxel pharmacokinetics. Overall, no large RCTs demonstrate any benefits of lycopene on tumour response, survival or toxicity reduction. Future development should focus on prevention, perioperative biomarker modulation, treatment development, and toxicity protection at specific doses and PK parameters, using biomarkers, appropriate controls, robust statistics, and meaningful endpoints. Together, these studies show that lycopene can be delivered to human plasma and prostate tissue and is well tolerated under these conditions. However, the literature is dominated by short-term prostate cancer studies, exploratory biomarkers, varied dietary and supplemental formulations, and small, unsupervised and/or poorly controlled trials (Table 2a–e). Food matrices and formulations induce different tissue concentrations, and dose alone cannot be reliably correlated with them. Existing evidence thus indicates exposure and feasibility, but therapeutic efficacy remains unknown in the prevention and treatment of cancer.

## 5. Synergistic and Nanodelivery Aspects

Some key properties of lycopene, such as extreme hydrophobicity, chemical instability, and rapid systemic clearance, limit its clinical utility as monotherapy and warrant novel approaches to overcome these challenges [82,83]. Recent research has focused on nanoscale delivery systems designed to enhance solubility and facilitate site-specific accumulation, thereby overcoming these pharmacokinetic barriers [83]. Cubosomal nanoparticles have emerged as a versatile platform. Researchers have engineered triphenylphosphonium (TPP)-functionalized cubosomes for mitochondrial-targeted delivery of lycopene and 5-fluorouracil, using the lipophilic TPP cation to exploit the mitochondrial membrane potential [84]. Similarly, lycopene-loaded niosomes conjugated to prostate-specific membrane antigen (PSMA) antibodies have shown superior cellular association and enhanced apoptotic activation in PSMA-positive prostate cancer cells compared to non-targeted formulations [85]. Other innovative systems, such as nanomicelles, have been shown to improve bioavailability by up to 2.2-fold and to modulate the NF-κB pathway, thereby decreasing tumour proliferation [86,87]. Similarly, nanostructured lipid carriers (NLCs) have been reported to provide sustained release and improved cytotoxicity in breast cancer models [88]. Preclinical studies suggest that lycopene may modify sensitivity to selected anticancer agents, including cisplatin and 5-fluorouracil. However, these findings are model-, dose-, and formulation-dependent and should not be interpreted as evidence of clinically established chemosensitisation or toxicity reduction [89]. In oral squamous cell carcinoma (OSCC), lycopene enhances cisplatin sensitivity by inhibiting MRP-1-mediated drug efflux and suppressing the PI3K/Akt/mTOR signalling axis, which simultaneously reverses epithelial–mesenchymal transition (EMT) [29,74,90]. The available lycopene–docetaxel investigation was a single-arm phase I dose-finding and pharmacokinetic study in patients with metastatic prostate cancer. It evaluated tolerability, dose selection, pharmacokinetic parameters, and exploratory biomarkers and was not designed or controlled to demonstrate improved prostate-specific antigen response, progression-free survival, or overall survival. Consequently, it cannot support a claim of superior clinical efficacy compared with standard chemotherapy [29,74]. Furthermore, lycopene, when combined with other phytochemicals, such as curcumin, exhibits synergistic effects in suppressing proliferation markers, including Ki-67, and pro-inflammatory cytokines, including TNF and IL-6 [91,92]. Integrated delivery systems, such as gellan gum-stabilised emulsions for co-delivering lycopene and quercetin, have been shown to maximise antioxidant synergy and bioavailability, offering a robust framework for multi-targeted, nutrition-based cancer therapies [93,94]. Early clinical evidence regarding the combination of lycopene and docetaxel remains preliminary. A small, single-arm phase II study enrolled 13 chemotherapy-naïve patients with metastatic castration-resistant prostate cancer who received docetaxel at 75 mg/m^2^ every 21 days together with oral lycopene at 30 mg/day. A reduction of at least 50% in prostate-specific antigen was reported in 10 of 13 patients (76.9% response rate), and the median overall survival was 35.1 months. However, the study did not include a concurrent docetaxel-only control group, and its comparison with previously published docetaxel outcomes was historical. Therefore, the study cannot establish that adding lycopene significantly improved prostate-specific antigen response or overall survival [74]. A separate 2024 phase I dose-escalation study evaluated synthetic lycopene at 30, 90, or 150 mg/day in combination with docetaxel and androgen-deprivation therapy in 24 patients with metastatic prostate cancer. The study primarily assessed safety, maximum tolerated dose, pharmacokinetics, and exploratory biomarkers. Although 150 mg/day was identified as the maximum tolerated dose and small changes in docetaxel exposure and selected biomarkers were observed, the trial was not designed or controlled to demonstrate improved treatment response, progression-free survival, or overall survival. The lycopene–docetaxel combination should therefore remain classified as investigational pending adequately powered randomised controlled trials [74]. These developments indicate that integrating nanotechnology with combinatorial regimens is needed to optimise lycopene’s therapeutic potential in modern oncology (Figure 3) [82,95]. Research on nanodelivery systems focuses on enhancing dispersion, reducing the effects of lycopene oxidation, improving loading efficiency, optimising lycopene release kinetics, improving cell uptake efficiency, and enhancing lycopene targeting [96]. Assessment of nanocarrier performance should encompass three interconnected aspects: physicochemical characteristics, biological validation, and translational readiness [97]. Physicochemical properties such as particle size and size distribution, surface charge, encapsulation efficiency, drug loading, stability, and drug release will be important [98]. When using biological assessment, focus on specificity, cellular uptake, applicable models (cancer and non-malignant), processing method, pharmacokinetics, biodistribution, and toxicity. Additionally, translational evaluation should assess manufacturing reproducibility, sterilisation, scale-up, batch consistency, and formulation stability and activity compared with controls [98,99]. Triphenylphosphonium-functionalized cubosomes appear to be a promising approach for the co-delivery of lycopene and 5-fluorouracil to mitochondria. The binding of PSMA-antibody-conjugated niosomes to prostate cancer cells expressing PSMA (input factors) is shown. These systems are primarily proof-of-concept and have not been tested for human pharmacokinetics, biodistribution, or systemic toxicity. According to cubosome data, the particle size is approximately 158.7 nm, the polydispersity index is 0.2482, and the encapsulation efficiencies of 5-fluorouracil and lycopene are ~79% and ~85%, respectively; these values should be confirmed before publication [84]. Lycopene- and Indocyanine green-co-loaded niosomes had an average hydrodynamic diameter of around 285 nm and encapsulation efficiencies of ~65% and ~45% for lycopene and Indocyanine green, respectively. An in vitro study with PSMA-expressing prostate cancer cells found that the PSMA antibody conjugates showed increased association and apoptotic activity. It did not validate tumour targeting, pharmacokinetics, immunogenicity or overall safety in vivo [100]. The mean particle size, zeta potential, EE, and DL of the nanostructured lipid carrier (NLC) formulation developed for oral delivery of lycopene were −121.9 nm, −29.0 mV, 84.5%, and 9.54%, respectively. The release profile was an initial burst over 4–9 h, followed by sustained release over 48 h. It showed better ex vivo permeation and in vitro cytotoxicity than lycopene without formulation, but the authors did not investigate in vivo pharmacokinetics or tumour biodistribution [101]. The results showed that the polymeric lycopene nanoparticles had a smaller diameter (62.10 nm) and higher encapsulation efficiency (91.84%) than in prostate cancer models. Following this, the system released 46.67% of the encapsulated compound after 24 h, similar to that obtained with free lycopene (26.19%). Efficacy testing has been conducted in LNCaP and PC-3 cell lines, but animal pharmacology, tumour response, and toxicity studies are lacking [102]. These findings were similar to pharmacokinetic data for micelles and chylomicrons in a non-cancer-specific context. The mean size and encapsulation efficiency of micelles were 7.5 nm and 78%, respectively, while chylomicrons were 131.5 nm with 80% efficiency. It was found that the bioavailability of lycopene in rats was low–6.8% for micelles and 9.5% for chylomicrons, indicating the possibility of changing systemic exposure by changing the carrier design and proving its anticancer activity [103]. More complex preclinical targeted cancer systems include folate-conjugated PEG-PLGA nanoparticles suspended in PEG. The mean particle size was 293.1 nm, the encapsulation efficiency was 72.63%, the average diameter was 0.247, and the lycopene loading level was 31.67%. Bleach released 93.11% over 168 h. They showed activity in the MCF-7 breast cancer model and non-malignant MCF-10A cells, as well as a rat-derived model of mammary gland malignancy after i.v. injection; they did not report any pharmacokinetic, biodistribution, ligand density, immunogenicity, or manufacturing scalability data [104].

## 6. Safety Aspects

Dietary sources of lycopene are usually safe. However, the safety profile of dietary exposure cannot be directly extrapolated to pharmacologic doses, longer-term use for cancer therapy, or combination with other drugs, such as chemotherapy and nanocarrier formulations [105,106]. The EFSA has established an ADI of 0.5 mg/kg b.wt/day, indicating that exposure via natural food sources is generally not expected to cause concerns, even when combined with human consumption of food additives or dietary supplements, which should be of concern [107,108]. Extensive toxicological assessments have found high doses of synthetic lycopene (up to 3000 mg/kg/day) to be safe with regard to behavioural changes and haematological or histopathological abnormalities in rodent subjects [76,109]. The acceptable daily intake is a food-safety reference value. It should not be interpreted as an oncology treatment dose or as evidence that pharmacologically administered lycopene is universally non-toxic. The ADI is a food-safety reference value and should not be considered the dose used to treat oncological disease, nor should it be taken to mean that all forms of pharmacologically administered lycopene are safe to take. Clinical trials have found lycopene to be safe. Supranutritional doses (up to 150 mg/day), even when used in oncology patients, were reported as well tolerated, with most adverse events being mild gastrointestinal side effects [80,110]. Additionally, lycopene exhibited cytoprotective and detoxifying effects against various endogenous and exogenous stressors, underscoring its safety profile. In rat models, lycopene-based formulations have demonstrated protective effects against experimentally induced toxicity. However, these findings are preclinical and cannot be directly extrapolated to human clinical outcomes. Further studies are required to determine whether similar biological effects are relevant under human exposure conditions. Lycopene can effectively reduce the systemic toxicity of regular chemotherapeutic agents. Lycopene administration has been reported to decrease cisplatin-induced nephrotoxicity and cardiotoxicity by activating the Nrf2 antioxidant protection system and lowering the expression of pro-apoptotic genes such as Bax and caspase-3 [111,112]. Further, recent research shows that lycopene can help protect major organs from damage induced by environmental toxicants such as hexavalent chromium. Mechanistically, lycopene regulates PINK1/Parkin-mediated mitophagy (mito-cleaning) and phthalates (e.g., DEHP) by reducing mitochondrial dysfunction and neuroinflammation [113,114]. Lycopene has also demonstrated protective effects against oxidative stress-induced tissue injury in mammalian models. In a rat model of thermal injury, lycopene supplementation reduced oxidative damage, inflammatory responses, and caspase-3 activity, suggesting its potential to modulate stress-induced apoptotic pathways [115]. However, certain restrictions also apply to high-dose lycopene. Excessive consumption may result in lycopenodermia, a harmless yellowish or orange tint to the skin that will eventually fade [116]. Experimental evidence suggests that other biological environments may show more significant, and potentially more detrimental, interactions. In a chronic alcohol-fed rodent model, high-dose lycopene supplementation was reported to increase hepatic cytochrome P450 2E1 (CYP2E1) expression and inflammatory foci, suggesting that lycopene’s biological effects may depend on dose, physiological context, and underlying disease conditions. These findings should be interpreted with caution and not directly extrapolated to human conditions [117]. Moreover, under intense UV radiation, lycopene may degrade into polar metabolites that may exhibit transient pro-oxidant behaviour. However, this is often offset by simultaneous induction and enhancement of endogenous antioxidant enzymes [118,119]. These findings emphasise the importance of adherence to proven ADI guidelines while reaffirming lycopene’s status as a biosafe candidate for long-term cancer prevention and adjuvant therapy [120,121].

## 7. Future Perspectives

Lycopene emerges as a versatile, multi-faceted bioactive molecule with a potential for an inclusive multi-pathway targeting of malignant cell survival. As detailed throughout this review, its ability to induce programmed cell death is not limited to a single signalling cascade. It also includes sophisticated modulation of the extrinsic (Fas–FasL) and intrinsic (Bcl-2/Bax) pathways, and targeted downregulation of oncogenic survival signalling pathways, including PI3K/Akt/mTOR and JAK-STAT3 [51,90,122]. Evidence for regulated cell-death pathways beyond apoptosis remains preliminary. Autophagy appears to be induced in some experimental systems and inhibited in others, whereas direct, lycopene-specific evidence for pyroptosis, necroptosis, ferroptosis, PANoptosome assembly, or PANoptosis remains insufficient. Future studies should use pathway-specific activation markers, dynamic flux measurements, genetic perturbation, pharmacological rescue, and exposure-confirmed tumour models before considering these mechanisms established [30,123]. The main obstacles to translating lycopene to the bedside are its physicochemical instability and low bioavailability [11,83]. Future research should improve and standardise nanoscale delivery systems, including cubosomes, nanomicelles, and targeted lipid liquid crystal nanoparticles (LLCs). They can deliver lycopene, in some cases by several-fold, to increase bioavailability and enable mitochondrial-targeted delivery [84,87,124]. Further developments are needed to ensure that therapeutic concentrations remain in the TME while avoiding systemic side effects. Future clinical development of lycopene should incorporate nutrigenomic and biomarker-guided approaches while avoiding premature claims of personalised therapeutic benefit. Variability in lycopene response among individuals may be influenced by baseline dietary exposure, formulation-specific pharmacokinetics, tissue distribution, and genetic variation in carotenoid absorption, transport, and metabolism [125,126]. For lycopene, future studies should define the intended clinical setting, standardise the formulation and dose, confirm plasma and tumour-tissue exposure, evaluate pharmacodynamic target engagement, and prospectively determine whether candidate genetic or metabolic markers predict treatment response. At present, these genetic and metabolic variables remain exploratory stratification factors rather than validated predictive biomarkers. Therefore, personalised lycopene-based oncology should be regarded as a future research framework rather than an established clinical strategy. In addition, lycopene’s anti-metaplastic effect, through its ability to sensitise cells to apoptosis, alongside its role in reducing chemotherapeutic side effects such as cardiotoxicity and nephrotoxicity, offers new strategies to chemosensitise tumours while protecting them from harmful drug side effects [2,74,83,112]. Overall, the apoptotic effect of lycopene has been established in vitro and in vivo, and extensive, standardised clinical trials (both large and small) are needed to establish this in humans [2,83]. Future research should go beyond basic pathway analysis to identify lycopene’s direct molecular interactors and targets. Multi-omics, AI-driven prediction, and chemistry strategies can help map lycopene’s molecular networks and biological effects. Emerging protein degradation technologies, such as PROTAC, combined with computational and experimental validation, offer promising avenues for exploring disease targets and deepening understanding of bioactive compounds [3,127]. As a result, lycopene can be a powerful, non-toxic adjuvant in the contemporary arsenal for treating cancer, once characterised as a biological phenotype. With advanced nanotechnology and a comprehensive understanding of individual metabolic phenotypes, lycopene could be optimised as an effective, non-toxic cancer therapeutic agent in today’s pharmacological arsenal.

## 8. Conclusions

Lycopene has a credible preclinical signal for modulating mitochondrial apoptosis and selected cancer survival pathways, particularly the BCL-2/BAX-caspase axis and PI3K/AKT/mTOR-related signalling. The evidence for Fas/FasL signalling is comparatively limited; JAK/STAT3 modulation is indirect and/or context-specific; and autophagy can be induced or inhibited depending on the experimental model, exposure, and assay. There is currently insufficient evidence that lycopene effects trigger PANoptosis. Biological exposure has been confirmed in human studies, and lycopene has been largely accepted as tolerable; however, these studies are limited in size and diversity and are largely confined to prostate cancer. These findings do not make lycopene a cancer therapeutic. Selected formulation characteristics and preclinical endpoints have been improved, but comparative pharmacokinetics and biodistribution, manufacturability, interaction potential, and clinical safety are poorly characterised. Thus, lycopene is a candidate investigational agent for cancer prevention or adjunctive therapy, not a proven agent. It will be clinically significant to evaluate standardised formulations, confirmed exposure pharmacology, biomarker-driven development, and drug-interaction profiling, and, if appropriate, to conduct adequately powered randomised clinical trials.

## Figures and Tables

**Figure 1:**
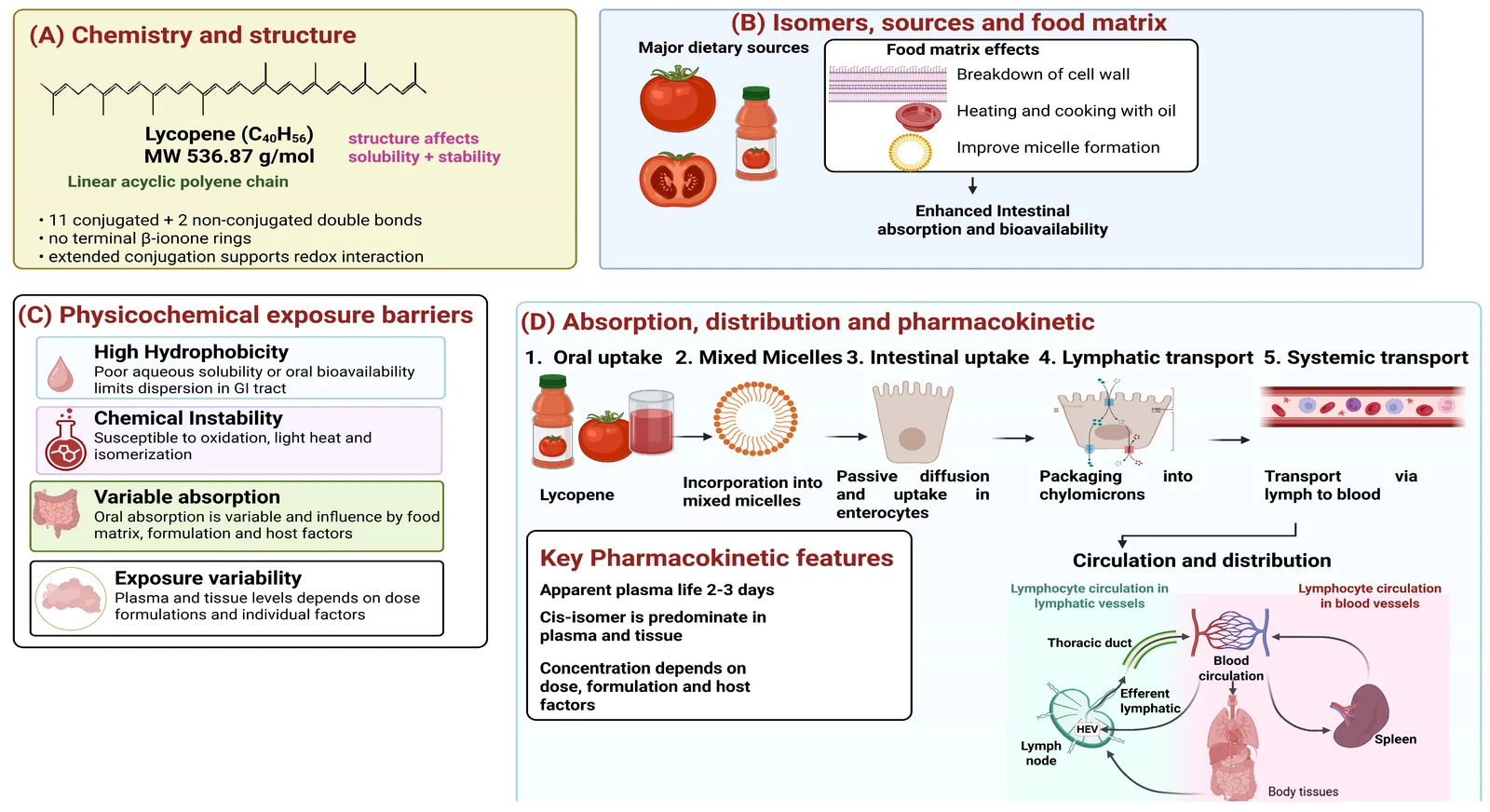
Chemical structure, isomeric forms, physicochemical properties, natural sources, and pharmacokinetics of lycopene. Created in BioRender. RAHMANI, A. H. (2026) https://BioRender.com/8rpm5ub.

**Figure 3:**
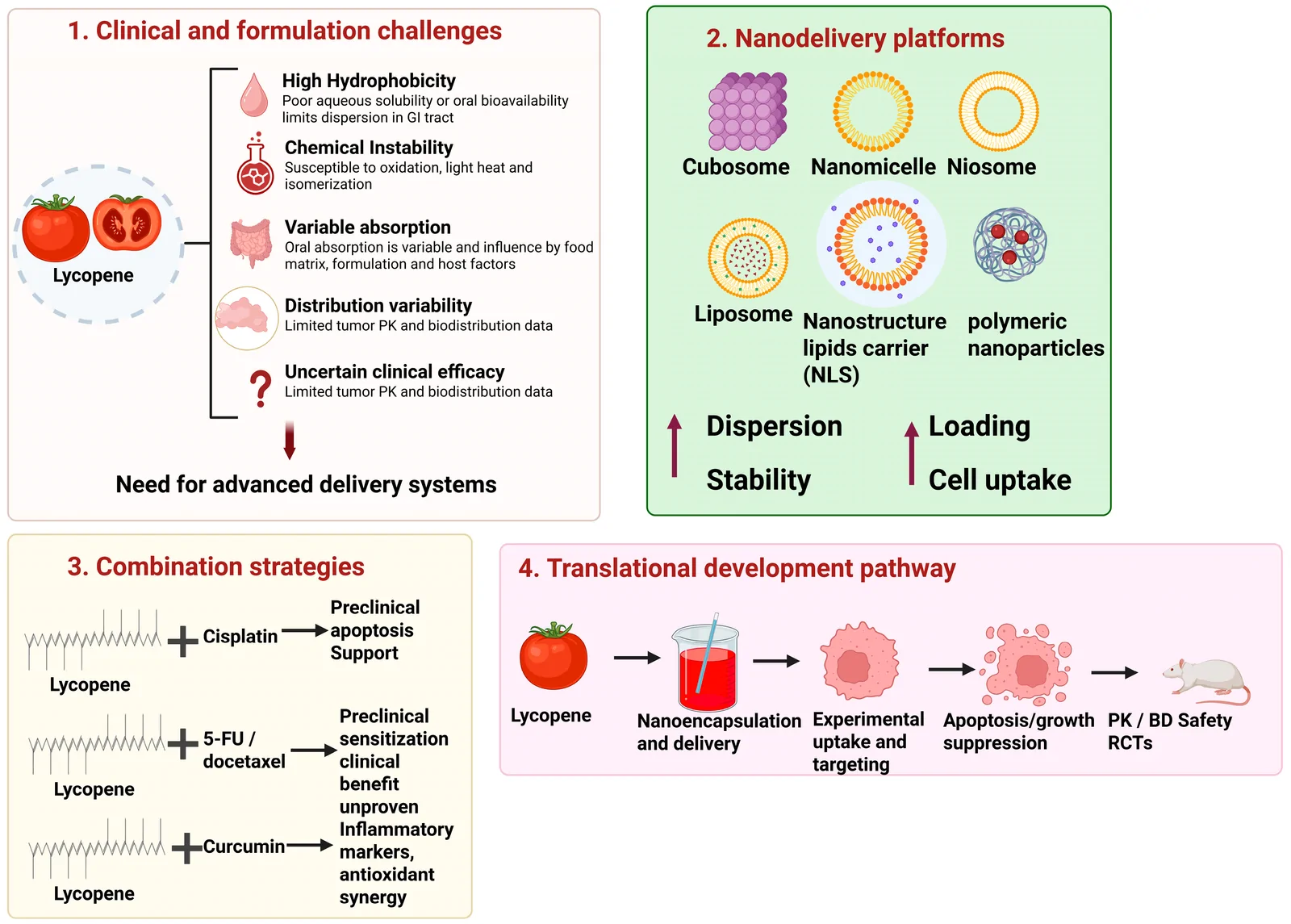
Synergistic and nanodelivery strategies for improving the anticancer potential of lycopene. Created in BioRender. RAHMANI, A. H. (2026) https://BioRender.com/8rpm5ub.

**Table 2:** (**a**). Human studies and clinical evidence relevant to lycopene and cancer. (**b**). Human studies and clinical evidence relevant to lycopene and cancer. (**c**). Human studies and clinical evidence relevant to lycopene and cancer. (**d**). Human studies and clinical evidence relevant to lycopene and cancer. (**e**). Human studies and clinical evidence relevant to lycopene and cancer.

(**a**)								
**Population and Cancer Setting**	**Lycopene Source or Formulation**	**Dose and Duration**	**Comparator**	**Main Endpoints**	**Main Findings**	**Exposure Information**	**Principal Limitations**	**References**
49,898 men; prostate cancer incidence and lethal prostate cancer	Dietary lycopene and tomato-product intake	Repeated dietary assessment during long-term follow-up	Categories of dietary exposure	Prostate cancer incidence, lethal disease, tumour angiogenic features	Higher lycopene exposure was associated with a lower risk of lethal prostate cancer and a less angiogenic tumour phenotype	Exposure estimated mainly from dietary intake and circulating measures	Observational design; residual confounding; dietary measurement error; supports prevention hypothesis, not treatment efficacy	[75]
26 men with localised prostate cancer	Tomato oleoresin extract	15 mg twice daily, equivalent to 30 mg/day, for 3 weeks	No supplementation	Plasma and prostate lycopene, PSA, tumour features, IGF-related biomarkers, BAX/BCL-2 and connexin 43	Increased biological exposure and exploratory favourable findings were reported; BAX and BCL-2 differences were not definitive.	Prostate-tissue exposure was measured after surgery	Small sample; short duration; open control; multiple exploratory endpoints; not powered for recurrence or survival	[76]
(**b**)								
**Population and Cancer Setting**	**Lycopene Source or Formulation**	**Dose and Duration**	**Comparator**	**Main Endpoints**	**Main Findings**	**Exposure Information**	**Principal Limitations**	**References**
32 men with localised prostate cancer; additional nonrandomized prostatectomy controls in the apoptosis analysis	Tomato sauce-based pasta meals	Approximately 30 mg/day for 3 weeks	Baseline comparison and nonrandomized surgical comparison group	Plasma and prostate lycopene, PSA, oxidative DNA damage, apoptosis, BAX and BCL-2	Increased tissue lycopene and changes in oxidative-damage and apoptosis-related markers were reported	Plasma and prostate lycopene were assessed	Nonrandomized or historical comparison; tomato-food matrix contains constituents other than lycopene; biomarker outcomes only	[65,77,78]
45 men with clinically localised prostate cancer	Lycopene supplement	15, 30, or 45 mg/day from biopsy to prostatectomy, approximately 30 days	No-supplement control	Safety, plasma lycopene, PSA, steroid hormones, sex hormone-binding globulin, tissue Ki-67	Exposure increased; selected hormone and Ki-67 changes were reported, particularly at the 30 mg dose	Plasma lycopene was measured; tumour exposure was not comprehensively related to response	Small dose groups; short duration; intermediate biomarkers; no recurrence or survival endpoint	[79]
(**c**)								
**Population and Cancer Setting**	**Lycopene Source or Formulation**	**Dose and Duration**	**Comparator**	**Main Endpoints**	**Main Findings**	**Exposure Information**	**Principal Limitations**	**References**
36 men with biochemical recurrence after definitive local prostate-cancer therapy	Oral lycopene supplement	15–120 mg/day for up to 1 year	No randomised comparator	PSA response, PSA progression, tolerability	No objective PSA responses were reported; PSA progression occurred in 37%; treatment was generally tolerated	Dose escalation was assessed, but tumour-tissue exposure was unavailable	Single-arm design; PSA surrogate endpoint; heterogeneous prior therapy; no control group or survival analysis	[80]
33 men scheduled for prostatectomy, predominantly localised prostate adenocarcinoma	Commercial tomato soup, sauce, or vegetable juice	Approximately 25–35 mg/day for 23 days	Lycopene-restricted diet of less than 5 mg/day	Plasma and prostate lycopene concentrations and isomer distribution	Plasma lycopene increased approximately 59–71%; prostate concentrations were 2.2–3.6-fold higher depending on the food source	Direct plasma and prostate-tissue measurement demonstrated source-dependent exposure	Small groups; exposure study rather than efficacy trial; prostate concentration was imperfectly predicted by plasma concentration	[72]
(**d**)								
**Population and Cancer Setting**	**Lycopene Source or Formulation**	**Dose and Duration**	**Comparator**	**Main Endpoints**	**Main Findings**	**Exposure Information**	**Principal Limitations**	**References**
79 patients with non-metastatic prostate cancer before curative treatment	Tomato products alone or tomato products plus several dietary components	Approximately 30 mg lycopene/day for 3 weeks	Control diet	Change in PSA and circulating nutritional biomarkers	No significant overall PSA difference; favourable signals were limited to exploratory subgroups and participants with greater biomarker increases	Plasma lycopene was measured	Short duration; food intervention; multi-component tomato-plus arm; subgroup findings were post hoc and require replication	[73]
13 chemotherapy-naïve patients with metastatic castration-resistant prostate cancer	Oral lycopene with intravenous docetaxel	Lycopene 30 mg/day; docetaxel 75 mg/m^2^ every 21 days	No concurrent docetaxel-only comparator	≥50% PSA reduction, time to PSA progression, response duration, overall survival	PSA reduction of at least 50% was reported in 10 of 13 patients; median overall survival was reported as 35.1 months	Lycopene exposure was not used to establish a dose–response relationship	Very small single-arm trial; historical comparison; cannot isolate the contribution of lycopene or establish added survival benefit	[81]
(**e**)								
**Population and Cancer Setting**	**Lycopene Source or Formulation**	**Dose and Duration**	**Comparator**	**Main Endpoints**	**Main Findings**	**Exposure Information**	**Principal Limitations**	**References**
24 men with metastatic prostate cancer	Synthetic oral lycopene with docetaxel and androgen-deprivation therapy	30, 90, or 150 mg/day during treatment	No randomised comparator	Safety, tolerated dose, docetaxel pharmacokinetics, angiogenesis and IGF-related biomarkers	A selected tolerated dose of 150 mg/day was identified; modest increases in docetaxel exposure and biomarker changes were observed	Docetaxel pharmacokinetics were directly measured	Phase I design; efficacy and survival not tested; interaction signal requires confirmation; adverse events occurred during combination therapy	[74]

## Data Availability

No new data were created or analyzed in this study. Data sharing is not applicable to this article.
